# Disruption of Intracellular Calcium Homeostasis as a Therapeutic Target Against *Trypanosoma cruzi*

**DOI:** 10.3389/fcimb.2020.00046

**Published:** 2020-02-14

**Authors:** Gustavo Benaim, Alberto E. Paniz-Mondolfi, Emilia Mia Sordillo, Nathalia Martinez-Sotillo

**Affiliations:** ^1^Instituto de Estudios Avanzados, Caracas, Venezuela; ^2^Facultad de Ciencias, Instituto de Biología Experimental, Universidad Central de Venezuela, Caracas, Venezuela; ^3^Department of Pathology, Molecular, and Cell-Based Medicine, Icahn School of Medicine at Mount Sinai, New York, NY, United States; ^4^Institute for Health Sciences, Mount Sinai St. Luke's & Mount Sinai West, New York, NY, United States

**Keywords:** trypanosomatids, calcium, new drugs candidates, signaling, therapeutic target

## Abstract

There is no effective cure for Chagas disease, which is caused by infection with the arthropod-borne parasite, *Trypanosoma cruzi*. In the search for new drugs to treat Chagas disease, potential therapeutic targets have been identified by exploiting the differences between the mechanisms involved in intracellular Ca^2+^ homeostasis, both in humans and in trypanosomatids. In the trypanosomatid, intracellular Ca^2+^ regulation requires the concerted action of three intracellular organelles, the endoplasmic reticulum, the single unique mitochondrion, and the acidocalcisomes. The single unique mitochondrion and the acidocalcisomes also play central roles in parasite bioenergetics. At the parasite plasma membrane, a Ca^2+^-^−^ATPase (PMCA) with significant differences from its human counterpart is responsible for Ca^2+^ extrusion; a distinctive sphingosine-activated Ca^2+^ channel controls Ca^2+^ entrance to the parasite interior. Several potential anti-trypansosomatid drugs have been demonstrated to modulate one or more of these mechanisms for Ca^2+^ regulation. The antiarrhythmic agent amiodarone and its derivatives have been shown to exert trypanocidal effects through the disruption of parasite Ca^2+^ homeostasis. Similarly, the amiodarone-derivative dronedarone disrupts Ca^2+^ homeostasis in *T. cruzi* epimastigotes, collapsing the mitochondrial membrane potential (ΔΨ_m_), and inducing a large increase in the intracellular Ca^2+^ concentration ([Ca^2+^]_i_) from this organelle and from the acidocalcisomes in the parasite cytoplasm. The same general mechanism has been demonstrated for SQ109, a new anti-tuberculosis drug with potent trypanocidal effect. Miltefosine similarly induces a large increase in the [Ca^2+^]_i_ acting on the sphingosine-activated Ca^2+^ channel, the mitochondrion and acidocalcisomes. These examples, in conjunction with other evidence we review herein, strongly support targeting Ca^2+^ homeostasis as a strategy against Chagas disease.

## Introduction

At present, there are no approved, highly effective therapies against *Trypanosoma cruzi*. The strategic rationale for current investigative efforts has been directed by consideration of the biological differences between the parasite and the host (mammalian) cells. The basic premise of this approach recognizes that Ca^2+^ is an essential signaling messenger in all eukaryotic cells studied so far, including trypanosomatids, and that fluctuation of the intracellular Ca^2+^ concentration ([Ca^2+^]_i_) is finely regulated, by diverse mechanisms at the plasma membrane level and by intracellular organelles. It has been extensively demonstrated that the various mechanisms responsible for regulation of [Ca^2+^]_i_ in trypanosomatids differ in many important features, from those in the host counterpart. Disruption of intracellular Ca^2+^ homeostasis by any means is lethal for all mammalian cells, since this is a driver to apoptotic processes or to necrosis (Nicotera et al., 1992), and appears also to be the case in trypanosomatids (Benaim and Garcia, 2011), including *T. cruzi*.

This review will focus on the similarities and differences between the general homeostatic systems responsible for the regulation of the [Ca^2+^]_i_ present in *T. cruzi* and in humans, that promote the ability of anti-trypanosomatid drugs acting on Ca^2+^ homeostasis to selectively cause parasitic death while minimally affecting the human host.

## The Requirement for Intracellular Ca^2+^ Regulation in Primordial Cells and Its Role as an Essential Signal in Trypanosomatids

Calcium is the fifth most abundant element on the Earth's crust, and the third most abundant metal (Carafoli and Krebs, 2016). Consequently, from the beginning of life on Earth, cells have had to deal with the presence of high concentrations of calcium, with the added problem that most calcium salts possess low solubility. Similarly, intracellular calcium complicates the choice of phosphate compounds as energy currency and phosphate-based bioenergetics, due to the poor solubility of calcium phosphate salts. This has implications not only for adenosine triphosphate (ATP) as an energy currency, but also for pyrophosphate (PP_i_), an important alternative energy coin in *T. cruzi*, as we will discuss below. For this reason, early in evolution cells were forced to develop sophisticated mechanisms to maintain a very low concentration of Ca^2+^ in the cytoplasm (usually below 100 nanomolar). Accordingly, Ca^2+^ has largely been compartmentalized in intracellular organelles, in which its concentration is very similar to the millimolar range encountered outside the cell, i.e., 4 orders of magnitude higher than in the cytoplasm. The extreme difference between the concentration of Ca^2+^ in the cytoplasm and in the exterior *milieu*, is even more remarkable when considered in the context of ionic distributions predicted for other cations by the Nernst equation. The difference between the intracellular and the extracellular concentrations calculated is far larger for Ca^2+^ than for any other ion normally present inside the cells (e.g., Na^+^, K^+^, Mg^2+^, H^+^). Maintaining the difference is associated with a high energy cost. However, throughout evolution, cells have taken advantage of this large Ca^2+^ electrochemical gradient to use it as an essential signaling messenger. The role of Ca^2+^ in cell signaling has been widely recognized in all eukaryotic cells so far studied including *T. cruzi* (Benaim and Garcia, 2011; Docampo and Huang, 2015; Schoijet et al., 2019). In the next section we will summarize different cell functions regulated either directly or indirectly by Ca^2+^ ions in this parasite.

## Different Processes Regulated by Ca^2+^ in Trypanosomatids

The function of Ca^2+^ as a signaling messenger in trypanosomatids is well-documented (Table 1). For example, in *T. cruzi* and *T. brucei*, Ca^2+^ binding proteins are important for the adhesion of the flagellum to the cell body and for flagellar activity (Maldonado et al., 1997; Docampo and Huang, 2015). In *Crithidia oncopelti*, Ca^2+^ plays a similar role in controlling flagellar activity, the direction of its flagellar wave propagation, and therefore its displacement (Holwill and McGregor, 1976). Specifically, the direction of the wave in the axoneme of *C. oncopelti* has been shown to be dependent on the [Ca^2+^]_i_ (Surgue et al., 1988). A role for calmodulin in determining the wave direction in *C. oncopelti* has also been proposed (Surgue et al., 1988).

**Table 1:** Some Calcium effects in different trypanosomatids.

	**References**
Microtubule assembly in *T. brucei*	Dolan et al., 1986; Robinson et al., 1991
Flagellar movements in *C. oncopelti*	Holwill and McGregor, 1976; Surgue et al., 1988
Flagellar movements in *T. cruzi* and *T. brucei*	Maldonado et al., 1997; Docampo and Huang, 2015
Cellular differentiation in *H. samuelpessoai*	Thomas et al., 1981
Cellular differentiation in *T. cruzi* and *T. brucei*	Lammel et al., 1996; Cortez et al., 2003; Walker et al., 2014; Docampo and Huang, 2015;
Cellular differentiation in *L. donovani*	Morrow et al., 1981
Invasion of the host cell in *T. cruzi* and other trypanosomatids	Misra et al., 1991; Moreno et al., 1994; Yakubu et al., 1994; Lu et al., 1997; Ruiz et al., 1998; Huang et al., 2013
Macrophage interaction in *Leishmania* spp.	Moreira et al., 1996; Cunningham, 2002; Dey et al., 2006; Naderer et al., 2011; Walker et al., 2014
Growth and proliferation in *L. donovani* and *T. brucei*	Selvapandiyan et al., 2001, 2007; Docampo and Huang, 2015
Nitric oxide transduction pathway in *T. cruzi*	Paveto et al., 1995
Osmoregulation in *T. cruzi*	Rohloff et al., 2003
Variant surface glycoprotein (VSG) release in *T. brucei*	Voorheis et al., 1982
Plasma membrane Ca^2+^-ATPase (PMCA) in different trypanosomatids	Benaim and Romero, 1990; Benaim et al., 1991, 1993a,b, 1995, 2013; Perez-Gordones et al., 2017; Ramírez-Iglesias et al., 2018
Calmodulin (CaM) in different trypanosomatids	Ruben et al., 1983; Benaim et al., 1987, 1995, 1998; Chung and Swindel, 1990; Benaim and Villalobo, 2002; Salas et al., 2005; Garcia-Marchan et al., 2009; Perez-Gordones et al., 2017
CaM stimulation of cAMP-phosphodiesterase in *T. cruzi*	Téllez-iñón et al., 1985
Ca^2+^-CaM Dependent protein kinase in *T. cruzi*	Ogueta et al., 1994, 1996, 1998
Calcium-stimulated adenylyl cyclase	D'Angelo et al., 2002
Flagellar Ca^2+^ binding protein	Engman et al., 1989

Ca^2+^ also plays an important role in the infectivity of several trypanosomatids, by increasing their capacity to invade host-cells. A transient [Ca^2+^]_I_ increase has been observed in trypomastigotes of *T. cruzi* (Yakubu et al., 1994) or amastigotes of *L. amazonensis* during their interaction with the host cell (Docampo and Huang, 2015), and in *L. donovani* during infection of macrophages (Misra et al., 1991). Furthermore, in the case of *T. cruzi*, bloodstream trypomastigotes invade the cells through a set of Ca^2+^-mediated interactions that trigger a signaling cascade in both the host cell and the parasite (Cortez et al., 2003; Walker et al., 2014). Ca^2+^ signaling through receptors for IP_3_, TcIP_3_R, and TbIP_3_R (see below) has been shown to modulate proliferation in *T. cruzi* (Docampo and Huang, 2015) and *T. brucei* (Huang et al., 2013), both *in vivo* and *in vitro*, as well as the cellular differentiation of these parasites.

The relationship between invasion by *T. cruzi* trypomastigotes and increased [Ca^2+^]_i_ in the parasite was first demonstrated during *in vitro* infection of L6E9 myoblasts monolayers (Moreno et al., 1994). After association with the myoblasts, parasite [Ca^2+^]_i_ increased from 20–30 to 340 nM; this increase was not observed in parasites that were not associated with myoblasts. Pretreatment of the parasites with Ca^2+^ chelators resulted in up to a 63% decreased in their ability to invade the myoblasts. A similar decrease was observed after addition of the chelating agent ethylene glycol tetraacetic acid (EGTA) to the host cell cultures, reducing the infective capacity of *T. cruzi* to 72 % (Moreno et al., 1994).

The increase in [Ca^2+^]_i_ in the host cell has been attributed to the expression of two glycoprotein membrane receptors expressed at the surface of the metacyclic trypomastigotes, gp82 and, to a lesser extent, the gp35/50 mucin-like protein. These parasites receptors mediate host Ca^2+^ signaling as a result of the contact between *T. cruzi* and the mammalian host cell (Burleigh and Andrews, 1998). Transient changes in [Ca^2+^]_i_ also appear to be necessary for the fusion of the host cell lysosome to the plasma membrane during the invasion of *T. cruzi* (Burleigh and Andrews, 1998). Furthermore, activation of the parasite tyrosine kinase proteins (PTK), which is also Ca^2+^-dependent, is involved in the internalization of *T. cruzi* in the host cell; while inhibition of parasite PTK activity decreases phosphorylation of the 175-kDa protein (p175) halting the ability of the parasites to enter the cells (Yoshida et al., 2000).

In *Leishmania* sp. a prolonged increase in the parasite [Ca^2+^]_i_, after invasion of mammalian cells, can trigger events that lead to parasite death by apoptosis (Moreira et al., 1996; Naderer et al., 2011). The uptake of Ca^2+^ by the parasite's organelles is essential for its thermotolerance between 34 and 37°, the temperature within the host cell. In addition, it has been suggested that the entry and regulation of Ca^2+^ and calcineurin signaling are necessary for the early and long-term adaptive responses of the parasite to environmental stressors found in the mammalian host (Naderer et al., 2011).

There is also evidence that Ca^2+^ signaling influences the differentiation of *T. cruzi* epimastigotes into metacyclic trypomastigotes through changes in cytosolic Ca^2+^ observed during this process (Lammel et al., 1996; Docampo and Huang, 2015). Ca^2+^ signaling participates in cell differentiation in *Herpetomona samuelpessoai* in a similar fashion (Thomas et al., 1981). Likewise, changes in the cytosolic Ca^2+^ levels are observed during the differentiation of the procyclic stages of *T. brucei* in the bloodstream (Walker et al., 2014).

In *L. donovani*, Ca^2+^ signaling has been shown to participate in the differentiation of the amastigote stage to promastigote (Morrow et al., 1981). Also in *L. donovani*, its cysteine Ca^2+^ -dependent protease caldonopain, a relative of calpain, has been shown to have a key role in catabolism, endogenous protein processing, cell invasion, and other biological actions (Dey et al., 2006). Moreover, these proteases are involved in the host-parasite interaction. The proteolytic activity of cytosolic caldonopain has been shown to be elevated in the presence of Ca^2+^ at the time of infection, and has been demonstrated to be involved in the metabolic turnover of intracellular proteins. Caldonopain activity may be essential for parasite survival during infection, to maintain intracellular Ca^2+^ homeostasis, and may play an important role in the signal transduction pathway (Dey et al., 2006).

One way in which promastigotes of *L. donovani* have been found to evade host defenses is by inhibiting fusion between the host cell phagosome and the endosome. The promastigotes alter their lipophosphoglycans molecules (LPG) by reducing the fusogenic properties of the membrane (Cunningham, 2002). In general, LPG, which is highly expressed on the surface of metacyclic promastigotes, interferes with the insertion of the membrane attack complex, and promastigote specific kinases deactivate the classical complement pathway (Walker et al., 2014). Once the parasite has passed into its amastigote phase, chelation of Ca^2+^ by LPG acts to protect the parasite within the phagolysosome. Ca^2+^ can bind to LPG repeating units near phosphate groups without altering glycan structure and the altered Ca^2+^ mobilization can lead to disturbed signal transduction, which in turn can drive defective PKC activation, thus increasing survival (Cunningham, 2002).

Cell growth and proliferation also appear to correlate with the parasite [Ca^2+^]_i_. In *L. donovani*, the centrins, which are Ca^2+^-binding proteins, directly influence the rate of parasite growth, which is exponential when parasite [Ca^2+^]_i_ is high, and stationary when the level is low. Of note, parasite knockout mutants lacking centrin show selective growth arrest of axenic amastigotes but not promastigotes (Selvapandiyan et al., 2001). In *T. brucei* centrins are involved in the segregation of organelles, coordination of nuclear and cellular division, and flagellar motility (Selvapandiyan et al., 2007; Docampo and Huang, 2015). On the other hand, *T. brucei* possesses an inositol 1,4,5-trisphosphate receptor (TbIP_3_R) in its acidocalcisomes involved in the Ca^2+^ signaling pathways. It has been shown that the presence of this receptor is necessary for the growth and establishment of infection of these organisms (Huang et al., 2013).

In *T. brucei*, the presence of Ca^2+^ is required for firm attachment of the microtubule-associated protein (MAP) p41 to the cytoskeleton, as well as for successful assembly of dimers and microtubular assembly. Even though p41 is normally found bound to cytoskeleton and preassembled microtubules of trypanosome tubulins, it remains tightly bound when calcium ions are present (Robinson et al., 1991). It has been known that Ca^2+^ initiates selective and complete depolymerization of the microtubules of *Trypanosoma brucei*, which supports the fact that this cation actively participates in the microtubular assembly (Dolan et al., 1986).

In addition, Ca^2+^ signaling also participates in osmoregulation in *T. cruzi* (Docampo and Huang, 2015), and in the transduction routes of nitric oxide in *T. cruzi* (Paveto et al., 1995). Ca^2+^ also plays an important role in the variant surface glycoproteins (VSG) release in *T. brucei* (Voorheis et al., 1982).

## Intracellular Ca^2+^ Regulation in Human Cells and Critical Differences with Respect to *Trypanosoma Cruzi*

*Trypanosoma cruzi* parasites must confront extreme changes in the extracellular Ca^2+^ concentration during different life cycle stages. In epimatigotes and trypomastigotes inside the insect host, the extracellular Ca^2+^ concentration is in the millimolar range, but for the amastigotes inside cardiac muscle cells this concentration falls well-below the submicromolar range. In eukaryotic cells, in general, intracellular Ca^2+^ regulation is a *conditio sine qua non* for the function of this cation as a signaling molecule, being achieved by the concerted participation of several mechanisms located in intracellular organelles and at the plasma membrane.

**Figure 1:**
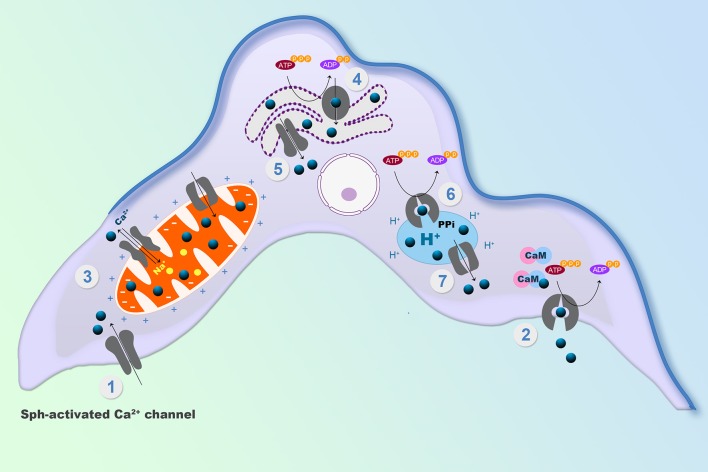
Schematic representation of the mechanisms involved in the intracellular Ca^2+^ regulation in *Trypanosoma cruzi*. (1) Sphingosine-activated Ca^2+^ channel, responsible for Ca^2+^ entry. (2) Calmodulin-regulated plasma membrane Ca^2+^ Pump, responsible for Ca^2+^ extrusion. (3) Mitochondrial Ca^2+^ Uniporter (MCU) and a Na^+^/ Ca^2+^ exchanger at the unique parasit mitochondrion. (4) SERCA type Ca^2+^ Pump at the endoplasmic reticulum and (5) a Ca^2+^ channel for Ca^2+^ release. (6) A PMCA type Ca^2+^-ATPase, responsible for Ca^2+^ accumulation in acidocalcisomes and (7) an IP_3_ Receptor for Ca^2+^ release from the acidocalcisomes to the cytoplasm (See text for detailed explanations).

Among the organelles in mammals, the endoplasmic reticulum and the mitochondria have central roles in reestablishing submicromolar Ca^2+^ levels in the cytoplasm, after any transient increases. In *T. cruzi*, there is, in addition, another very important system, the acidocalcisomes. Acidocalcisomes are acidic vacuoles loaded with Ca^2+^, polyphosphates and other ions, that are essential for viability in trypanosomatids, but which are only present in specialized cells (e.g., platelets, mast cells, basophils) in humans (Patel and Docampo, 2010; Huang et al., 2014). Accordingly, the acidocalcisomes are considered as a potential target for the action of drugs against these parasites (see below) (Figure 1). Mitochondria are able to accumulate Ca^2+^ in large quantities, through a so-called mitochondrial Ca^2+^ uniporter (MCU) that utilizes the electrochemical H^+^ gradient across the internal mitochondrial membrane (De Stefani et al., 2011) to dissipate energy as Ca^2+^ is accumulated (Figure 1). This MCU is widely conserved throughout evolution, being present in all mitochondria so far described, including the trypanosomatid single unique mitochondrion (Docampo and Vercesi, 1989; Benaim et al., 1990). The low affinity of the MCU for Ca^2+^ is such that for a long time it was not included as a true Ca^2+^ regulator. Instead, it was presumed to function as an import mechanism to fulfill the demands for Ca^2+^ of at least three different mitochondrial dehydrogenases. However, by utilizing the mitochondrial-targeted aequorin, a protein the emits light when bind to Ca^2+^, it was clearly demonstrated that the mitochondria indeed participate in Ca^2+^ signaling, not only in mammals (Rizzuto et al., 1992; Pozzan et al., 2003) but also in trypanosomatids (Xiong et al., 1997; Ramakrishnan and Docampo, 2018), since this organelle was able to flash when a particular signal increased the parasite [Ca^2+^]_i_. The explanation for these observations is that some mitochondria can physically approach Ca^2+^ channels in particular *loci* inside the cell, below the plasma membrane and near certain organelles where the locally-released Ca^2^ can reach concentrations compatible with the low affinity of the MCU (Benaim et al., 1990). This fact has allowed the triumphal return of the mitochondria to the signaling scenario. More recently, two subunits of the *Trypanosoma cruzi* MCU complex containing canonical EF hand domains (MICU1 and MICU2), have been studied by the use of the CRISPR/Cas9 system, demonstrating that albeit their overexpression does not significantly affect mitochondrial Ca^2+^ uptake, their ablation has a large effect on the cation uptake by the mitochondria supporting their role in the stabilization of the MCU complex (Bertolini et al., 2019). In the case of *T. brucei*, the MCU complex was found to possess two additional subunits not found in mammals named TbMCUc and TbMCUd, which are essential for mitochondrial Ca^2+^ uptake (Huang and Docampo, 2018). These two units have been identified and characterized in *T. cruzi* (TcMCUc and TcMCUd). By the use of the CRISPR/Cas9 system it has been shown that overexpression of these genes drives an increase of mitochondrial Ca^2+^ uptake. Conversely, knockout of any of these genes leads to a loss of Ca^2+^ uptake, although the mitochondrial membrane potential is maintained (Chiurillo et al., 2019). Since TcMCUc and TcMCUd are not present in mammals and are of significant importance in several functions in *T. cruzi*, mainly related to its bioenergetics, they represent an attractive alternative drug target against these parasites (Chiurillo et al., 2019).

The advanced application of the CRISPR/Cas9 system to investigation of *T. cruzi* biology (Lander and Chiurillo, 2019) has enormously facilitated the finding and characterization of many proteins in these parasites. The recent identification in *T. cruzi* of a mitochondrial pyruvate dehydrogenase phosphatase (TcPDP) that is sensitive to physiological Ca^2+^ concentration and is able to stimulate the activity of a mitochondrial pyruvate dehydrogenase, stimulating energy metabolism through the Krebs cycle activation, has provided further insight into the role of Ca^2+^ in *T. cruzi* bioenergetics (Lander et al., 2018). The activity of this enzyme was demonstrated to be required for *T. cruzi* growth, differentiation, and infectivity (Lander et al., 2018).

The endoplasmic reticulum possesses a Ca^2+^ pump, the sarco(endo)plasmic reticulum Ca^2+^-ATPase (SERCA) that accumulates large amounts of Ca^2+^ by transporting the cation at the expense of ATP hydrolysis, and has high Ca^2+^ affinity (Benaim and Garcia, 2011), enabling a high Ca^2+^ concentration inside this organelle, similar to the extracellular *milieu* (i.e., around 2 mM) (Figure 1).

The activity of the SERCA in mammalian cells is inhibited by tapsigargin and ciclopiazonic acid, and also by the sphingolipid sphingosine (Benaim et al., 2016). In *T. cruzi*, a Ca^2+^-ATPase (TcSCA) that localizes at the endoplasmic reticulum (ER), has been partially characterized (Furuya et al., 2001), and shown to possess several sequence motifs found in SERCA. Although TcSCA can be inhibited by ciclopíazonic acid, thapsigargin fails to inhibit the enzyme (Furuya et al., 2001); the effect of sphingosine has not been studied so far. In mammals, there are at least two Ca^2+^ channels in the ER, the IP_3_ Receptor (IP_3_R) and the Ryanodine receptor (RyR, more predominant in excitable cells), that allow the Ca^2+^ release to the cytoplasm when they are opened by a particular signal. The activity of IP_3_R is modulated by IP_3_, the product of the hydrolysis of PIP_2_ by several isoforms of PLC (Furuichi et al., 1989). By contrast, addition of IP_3_ fails to cause Ca^2+^ release in *T. cruzi*, although the machinery for the synthesis of IP_3_ has been well documented in trypanosomatids (Docampo and Pignataro, 1991). Interestingly, in trypanosomatids, the IP_3_ receptor appears to be present in acidocalcisomes (see below). These organelles possess several different transporters, pumps and exchangers, for the accumulation of Ca^2+^ and other cations, such as a Vacuolar type H^+^ -ATPase, a Ca^2+^-ATPase similar to that found at the plasma membrane (PMCA), as well as other distinct Ca^2+^ transport mechanisms (Huang et al., 2014). Notably, acidocalcisomes possesses a proton pumping pyrophosphatase (H^+^-PPase) that allows the accumulation of H^+^, and hence the acidification of this organelles. The name of the organelle is derived from this capability, together with its capacity for Ca^2+^ accumulation (Docampo and Huang, 2015). Acidocalcisomes possess large amounts of orthophosphate (P_i_), polyphosphates and particularly pyrophosphate (PP_i_), which is particularly important for *T. cruzi*, and other trypanosomatids and apicomplexan parasites (e.g., *Plasmodium spp*).

In contrast to their human hosts, which are dependent on hydrolysis of ATP, these parasites can use PP_i_, which has essentially the same free energy of hydrolysis of ATP, as an alternative energy currency (Docampo and Huang, 2015). Of course, this difference has been exploited in the development of a possible pharmaceutical target against Chagas disease, by the use of bisphosphonates, a group of molecules that are able to selectively inhibit pyrophosphatases (Montalvetti et al., 2001). Interestingly, H^+^-PPases have also been found at the plasma membrane and in the Golgi apparatus (Martínez et al., 2002), where the pyrophosphate analogs aminomethylenediphosphonate and imidodiphosphate block the acidification of plasma membrane vesicles in *T. cruzi*. This evidence demonstrates, as expected, that H^+^-PPases are not localized solely to acidocalcisomes, but are present in other *loci* that may utilize PPi as an energy source.

Substantial recent experimental evidence in *T. cruzi* supports localization of the IP_3_R in the acidocalcisome and not at the ER. Furthermore, experiments with CRISPR/Cas9 and other techniques (Lander et al., 2016) have demonstrated that the parasite IP_3_R is fully functional when IP_3_ is present. This receptor (TcIP_3_R) has been cloned, expressed, and associated with proliferation, differentiation virulence, and infectivity (Hashimoto et al., 2013), by the K. Mikoshiba Group, who first discover the IP_3_R (Furuichi et al., 1989). In that work immunofluorescent labeling was used to demonstrate the localization of this receptor to the ER, as for mammalian cells. However, it appears that observation may have been the result of over-expression, since the use of the CRISPR/Cas9 system for C-terminal tagging of genes in *T. cruzi* (Lander et al., 2016) allows the confirmation of acidocalcisomes as the organelles where IP_3_R was indeed present. The localization of this receptor in acidocalcisomes and the contractile vacuole complex, together with the differences in sequences, emphasizes an essential distinction between *T. cruzi* and its human counterpart that could be a useful consideration for a rational therapeutic development.

Because IP_3_R-mediated Ca^2+^ signaling is key to a plethora of cellular events in *T. cruzi* such as transformation and replication in its various developmental stages the potential use of receptor antagonists requiring this Ca^2+^ signaling cascade as well as TcIP_3_R inhibitors could emerge as potential therapeutic targets that could be of benefit from a clinical standpoint. Comparisons amongst human and *T. cruzi* IP3Rs primary structure may assist in identifying other likely inhibitory compounds by high-throughput or virtual screening of chemical libraries (Hashimoto et al., 2013).

**Figure 2:**
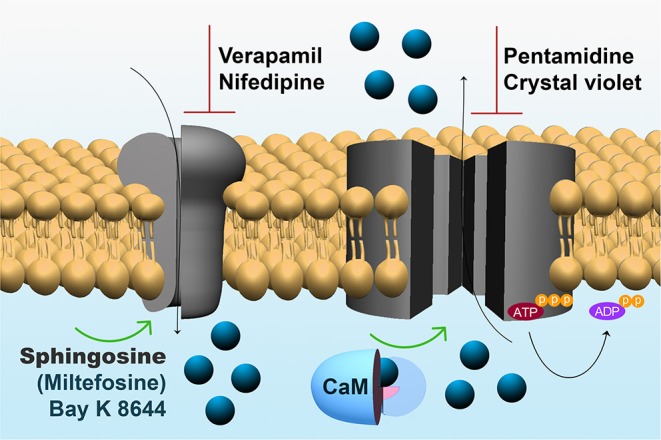
Expanded model of Ca^2+^ regulating mechanisms within the parasites plasma membrane. **Left:** The sphingosine-stimulated Ca^2+^ channel, where the activation by Bay K 8466 and miltefosine and the inhibition by nifedipine and verapamil are depicted. **Right:** The CaM-stimulated PMCA, where the inhibition by pentamidine and crystal violet are shown (See text for explanations).

The endoplasmic reticulum, the mitochondrion and acidocalcisomes act in concert whenever an elevation of the [Ca^2+^]_i_ has occurred, necessitating their participation to return the concentration to the cytoplasmic basal level. However, the capacity of these organelles is limited by their volume as compartments. Thus, the mechanisms of Ca^2+^ regulation located at the plasma membrane are responsible for the long-term regulation of the [Ca^2+^]_I_, since they, in principle, are able to extrude Ca^2+^ against a virtually infinite space (Figure 2). At the plasma membrane of human cells there are only two mechanisms described for Ca^2+^ extrusion, a Na^+^/Ca^2+^ exchanger present mainly in excitable cells, and a plasma membrane Ca^2+^-ATPase (PMCA), which has been identified in all eukaryotic cells so far described, including *T. cruzi* (Benaim et al., 1991). Attempts to identify a Na^+^/Ca^2+^ exchanger in trypanosomatids have been unsuccessful (Benaim et al., 1993a; Docampo and Huang, 2015), supporting the ubiquitousness of the PMCA. This Ca^2+^ pump has a very high affinity for Ca^2+^, and is stimulated by calmodulin (CaM), the also ubiquitous Ca^2+^-binding protein, present in all eukaryotic cells (Benaim and Villalobo, 2002). CaM increases the affinity of the enzyme for Ca^2+^ and ATP, also raises its V_max_ by a very well-known mechanism in humans (Benaim et al., 1984). This Ca^2+^-sensing protein binds to an auto-inhibitory CaM-binding domain removing it from the active site, thus increasing its affinity for their substrates, Ca^2+^ and ATP (Benaim et al., 1984). In *T. cruzi* the PMCA also has been identified (Benaim et al., 1991), isolated by mean of a CaM-affinity column and partially characterized (Benaim et al., 1995), but appears to diverge from its human counterpart at the CaM-binding domain (Figure 2). Albeit the CaM-binding domain in *T. cruzi* PMCA (TcCa1) has not been characterized fully, recent studies on *Trypanosoma equiperdum* (a *T. brucei*-related hemoflagellate parasite that causes infection in cattle), have demonstrated that the CaM-binding domain of the PMCA of *T. equiperdum* possesses a non-canonical sequence (Perez-Gordones et al., 2017; Ramírez-Iglesias et al., 2018). This Ca^2+^-ATPase contains a 28 amino acid-region in the C-terminal tail that has been proposed to assume an α-helix conformation within a 1–18 (Trp-1, Phe-18) CaM binding motif (Perez-Gordones et al., 2017). Another difference is that unlike CaM in human cells, which interacts with the CaM-binding domain solely with the C- terminal half (78–148 aa) of the PMCA protein (Guerini et al., 1984), *T. equiperdum* CaM appears to wrap the CaM-binding domain of the parasite PMCA (Perez-Gordones et al., 2017; Ramírez-Iglesias et al., 2018). The sequence of the CaM-binding domain of *T. equiperdum* is very similar to that present in the in the *T. cruzi* PMCA, suggesting that with all likelihood, the characterization performed on the enzyme from *T. equiperdum*, can be extrapolated to the *T. cruzi* PMCA. In fact, the CaM-Binding domain from *T. cruzi* PMCA, albeit bearing a 1–17 CaM binding motif instead of the 1–18 motif present in *T. equiperdum* (since there is a gap by the lack of one amino acid), can still form an α-helix predicted to be able to bind CaM (G. Benaim, unpublished observations).

The differences between human and *T. cruzi* CaM deserve special consideration, given that this ubiquitous Ca^2+^ binding protein has been highly-conserved in eukaryotes, with trypanosomatids being a remarkable exception. Although vertebrate and trypanosomatid CaMs all have 148 amino acids, and the structure of CaM is identical in all vertebrates, there are 16 amino acid substitutions between *T. cruzi* CaM when compared to vertebrate CaM (~89 % homology) (Garcia-Marchan et al., 2009). Trypanosomes contain three genes tandemly repeated for CaM, and this protein is highly expressed in these parasites (Chung and Swindel, 1990). We have calculated, based on an estimated volume of the *T. cruzi* epimastigote, and based on the assumption that the quantity of CaM obtained in the purification procedure is fully conservative (i.e., All the CaM from the batch was isolated), that the CaM concentration in trypanosomatids is about 2 μM (Benaim, G. Unpublished observations). This concentration is similar to that found in the mammalian tissues (i.e., brain and testis) in which the highest concentrations of this Ca^2+^-binding protein are found, emphasizing the critical importance of this protein in *T. cruzi*.

Vertebrate CaM is characterized by a Ca^2+^-shift when run on SDS-PAGE in the presence or absence of the calcium chelator EGTA (i.e., with or without Ca^2+^). This trademark of CaM is probably due to the increase in the α-helix content and the concomitantly increase in the hydrophobic character of the protein, or the acquisition of a more globular shape, even in the presence of SDS (Garcia-Marchan et al., 2009). Similarly, a Ca^2+^-shift also has been observed in *T. cruzi* CaM. However, albeit possessing the same molecular mass as the vertebrate CaM, *T. cruzi* CaM migrates more to the anode (lower apparent molecular mass), likely due to its more hydrophobic character. This is concordant with circular dichroism studies, which have confirmed that in the presence of Ca^2+^ the α-helix content of *T. cruzi* CaM is increased is comparison to mammal CaM (Garcia-Marchan et al., 2009).

At present, the full significance of these differences in the molecular structure of this important protein is not known. As mentioned, CaM is able to stimulate the *T. cruzi* PMCA; the affinity of the human PMCA for CaM is slightly lower than for *T. cruzi* CaM. In addition, the CaM-anatgonists trifluoperazine and calmidazolium, have been shown to have less inhibitory activity when the PMCA is stimulated by the *T. cruzi* CaM when compared to rat CaM (Garcia-Marchan et al., 2009). These findings raise the possibility that there is a still-undiscovered CaM target in this parasite that could reflect the prominent differences in structure encountered between the *T. cruzi* and vertebrate proteins.

On the other hand, it has been demonstrated that CaM phosphorylation may vary based on its target enzyme (Benaim and Villalobo, 2002; Salas et al., 2005). The composition of phosphorylatable aminoacids in *T. cruzi* CaM differs significantly from its vertebrate counterpart. For example, vertebrate CaM bears four serine and 12 threonine residues, whereas *T*. *cruzi* CaM contains nine serine and nine threonine residues; thus, varying the pattern of phosphorylation of this protein when phosphorylated by the epidermal growth factor receptor (EGFR) in its known preference for Tyr motifs.

Given that *T. cruzi* CaM possesses only one tyrosine (Tyr-138) residue lacking the Tyr-99 motif, the main target of the EGFR, low-level phosphorylation occurs, as opposed to phosphorylation on serines and or threonines, which are significantly larger *T. cruzi* CaM when compared to that of vertebrates (Benaim et al., 1998). How this finding may affect CaM activity and its possible functional repercussion on the parasites physiology is a point that deserves further investigative attention.

The mechanism responsible for Ca^2+^ entry in *T. cruzi* has been discovered just recently (Figure 1). A sphingosine-stimulated plasma membrane Ca^2+^-channel similar to that described in *L. mexicana* (Benaim et al., 2013) has been reported (Rodriguez-Duran et al., 2019), and characterized electrophysiologically by Patch Clamp techniques (Figure 2). This Ca^2+^ channel shares some characteristics with the human L-Type Voltage gated Ca^2+^ channel (VGCC), including inhibition by the VGCC blocker nifedipine, a dihydropiridine, and activation by Bay K8644, but differs from the L-type VGCC in its activation by sphingosine. Although there seems to be homology with the human orthologue VGCC, the *T. cruzi* channel differs in that it appears not to be voltage-dependent. Another remarkable difference from its human orthologue is its stimulation by miltefosine, a unique oral drug approved against visceral leishmaniasis, and also of potential use against Chagas disease (see below).

## Targeting Intracellular Ca^2+^ Homeostasis as a Strategy Against Chagas Disease

**Figure 3:**
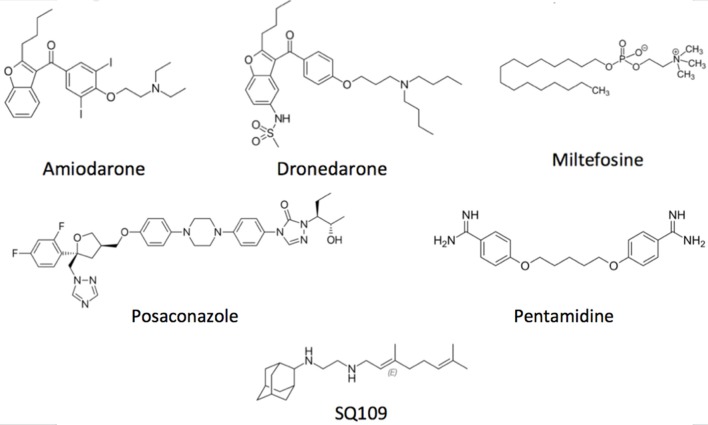
Structural depiction of main compounds known to exert trypanocidal effect through intracellular Ca^2+^ homeostasis disruption.

Concerning the main goal of this review, there are several drugs that produce their trypanocidal effect through disruption of parasite Ca^2+^ homeostasis. Amiodarone (Figure 3), a commonly used antiarrhythmic, has been shown to exert a potent effect directly on *T. cruzi*, by inducing a large increase in the [Ca^2+^]_i_ (Benaim et al., 2006). This effect was shown to be mediated by the release of the cation from intracellular compartments, since the effect was independent of the presence of calcium in the extracellular *milieu*. It was demonstrated that amiodarone acts directly on the mitochondrion, collapsing the electrochemical membrane potential of the parasite without affecting the host cell. This in turn induces rapid Ca^2+^ release to the cytoplasm (Benaim et al., 2006). Amiodarone also affects the acidocalcisomes, by inducing alkalinization, concomitantly with Ca^2+^ release. In concert, these effects induce the large intracellular Ca^2+^ elevation observed when *T. cruzi* is exposed to amiodarone. Similar results have been observed when dronedarone (Figure 3), another benzofuran derivative, was added instead of amiodarone to *T. cruzi* (Benaim et al., 2012). Dronedarone, which was synthesized to overcome the adverse effects of amiodarone due to the presence of iodine in its structure and its extremely hydrophobic character, also appears to be even more effective than amiodarone *in vitro* against *T. cruzi*. The effect of dronedarone on the *T. cruzi* mitochondrion and acidocalcisomes was more rapid than that of its predecessor amiodarone, and also resulted in a lower IC_50_ (0.75 μM) than did amiodarone (IC_50_ 2.7 μM) when determined on amastigotes inside mammalian host cells, the clinically relevant phase of the parasite (Benaim and Paniz-Mondolfi, 2012; Benaim et al., 2012). Interestingly, both antiarrhythmic were also very effective against *Leishmania mexicana*, a common causative agent of cutaneous leishmaniasis in the New World, demonstrating a very low IC_50_ on amastigotes inside macrophages (Serrano-Martín et al., 2009a,b; Benaim et al., 2014).

Importantly, amiodarone, when used in combination with miltefosine, induced parasitological cure of mice infected with *L. mexicana* (Serrano-Martín et al., 2009b). In cardiomyocytes, amiodarone directly acts on the recovery of F-actin fibrillar organization, connexin43 distribution, and the recovery of spontaneous contractility in *T. cruzi*-infected cardiac myocytes, simultaneously with the eradication of the infection (Adesse et al., 2011), further explaining and supporting the benefits of this drug *in vivo*. Amiodarone has been used successfully in a few cases for compassionate treatment of severe Chagas Disease in humans (Paniz-Mondolfi et al., 2009). Recently, evidence for the effectiveness of amiodarone *in vivo*, has been reported in a study trial including 105 infected privately- owned and military working dogs at Lackland Air Force Base in Texas (USA) (Madigan et al., 2019) in which a combination treatment of amiodarone and itraconazole –an ergosterol synthesis inhibitor- was used. In this trial survivability of treated canines increased to 95.3%, compared to 53% of untreated controls. Concomitantly, treated dogs experienced 98.2% clinical improvement, compared to 27% of controls (Madigan et al., 2019). Notably, amiodarone has already proved beneficial for suppression of complicated ventricular arrhythmias in chagasic patients. In fact, amiodarone's proven efficacy when combined with benznidazole was demonstrated in the BENEFIT trial (Morillo et al., 2015), albeit apparently overlooked.

Reported amiodarone cardiotoxicity is not related to Ca^2+^ homeostasis but rather to blockade of potassium channels, which may lead to significant bradycardia and marked prolongation of the QT interval, and ultimately to the induction of polymorphic ventricular arrhythmias (Colunga-Biancatelli et al., 2019). Nevertheless, such events are very rare and usually multifactorial, with amiodarone still exhibiting the lowest pro-arrhythmic action when compared to other antiarrhythmic agents. Allelic variants of the coding region of congenital long QT syndrome have been reported in up to 15% of patients with amiodarone induced QT prolongation, thus suggesting the role of a distinct predisposing genetic background (Hoffmann et al., 2012). Of note, amiodarone has been shown to contribute to full cardiomyocyte structural and functional recovery after treatment (Adesse et al., 2011). The restoration of connexin43 expression and distribution, and of spontaneous contractility in cradiomyocytes (Adesse et al., 2011) translates clinically into reduction of arrhythmogenic events. Altogether, these findings support further clinical evaluation of amiodarone as a potential treatment for Chagas disease.

More recently, a new benzofurane derivative with a construction design based on amiodarone's structure (Amioder) has been shown to display a potent effect on epimastigotes and cell-infected amastigotes from *T. cruzi* (Pinto-Martinez et al., 2018a). As expected, the mechanism of action, was similar to that of amiodarone, causing increased the [Ca^2+^]_i_, collapsing the electrochemical membrane potential of the mitochondrion, and alkalinizing the acidocalcisomes of the parasite. The same overall effect was also recently found by Amioder on *L. donovani* (Martinez-Sotillo et al., 2019).

**Figure 4:**
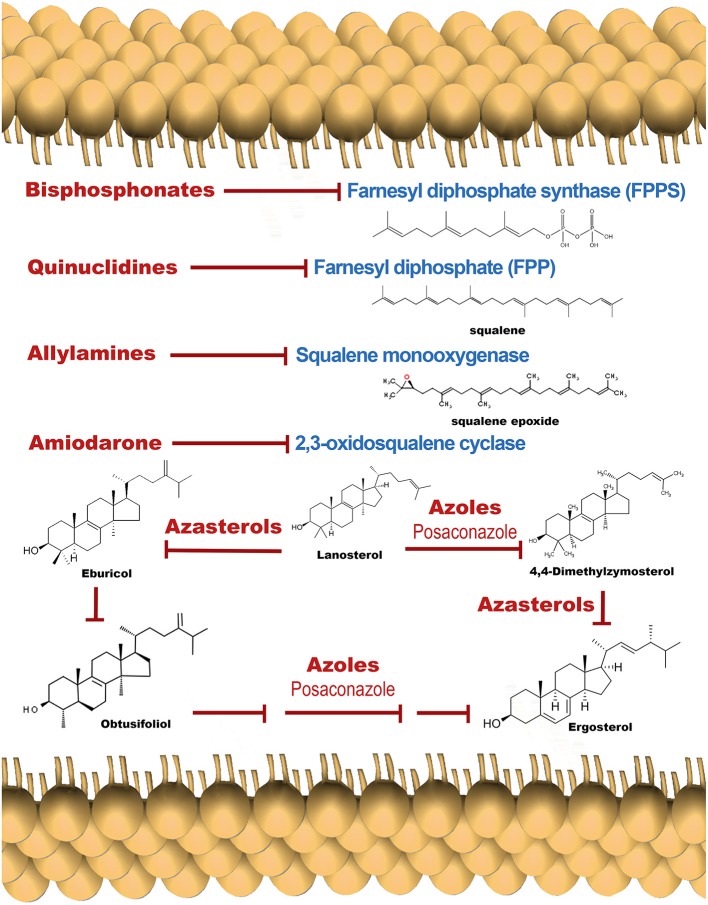
Schematic representation of the ergosterol synthesis pathway, showing target points for the main inhibitors.

Posaconazole (Figure 3), an inhibitor of ergosterol synthesis (Figure 4) acting on the enzyme C14-α-demethylase, and already approved as antimycotic, has also shown to exert a dramatic effect on *T. cruzi*, and has proven effective in humans treated under compassionate use. In one case, a woman with systemic erythematosus lupus who developed acute Chagas disease during immunosuppressive treatment (methylprednisolone, prednisone, and cyclophosphamide), was successfully treated with posaconazole (Pinazo et al., 2010). On the other hand, experimentally, posaconazole has shown to dramatically increase the basal intracellular Ca^2+^ levels in epimastigotes from *T. cruzi*, provided that the parasites have been allowed to be depleted of endogenous ergosterol, by incubation for 96 h in the presence of 12.5 nM of the drug (Benaim et al., 2006). Therefore, posaconazole can also be included as a potential emerging antichagasic drug that acts by perturbance of the intracellular Ca^2+^ homeostasis. However, despite displaying significant antitrypanosomal activity, results from the CHAGASAZOL trial indicated that the use of posaconazole resulted in a larger percentage of treatment failures than benznidazole (Molina et al., 2014). Benznidazole monotherapy was also shown to be superior to posaconazole in the STOP-CHAGAS trial (Morillo et al., 2017). Nevertheless, results of these trials should be examined carefully in the context of dosage, duration of treatment, and the nature of infecting parasite strain, since it has been demonstrated that these factors can influence treatment outcome. Dose adjustment and the use of novel delayed-release formulations with increased and sustained bioavailability will largely and positively influence treatment efficacy in future and ongoing trials (Urbina, 2017). By contrast, treatment with high dose posaconazole (400 mg BID) has proved successful for treatment of cutaneous leishmaniasis caused by *L. infan*tum in a human patient (Paniz-Mondolfi et al., 2011).

Interestingly, pentamidine (Figure 3), an aromatic diamidine compound introduced in 1940s is also frequently used for treating Sleeping Sickness caused by *T. brucei*, as well as leishmaniasis. Use of pentamidine has been also suggested for Chagas disease, since it blocks the transport of putrescine, a precursor of trypanothione in trypanostomids (Díaz et al., 2014). Among the multiple functions attributed to this drug in these parasites, pentamidine is able to selectively inhibit the plasma membrane Ca^2+^-ATPase (PMCA) activity and its associated Ca^2+^ transport in *T. brucei* (Benaim et al., 1993b), without affecting its human homologue enzyme. Instead, this drug behaves as a poor CaM antagonist (Benaim et al., 1993b).

Very similar results have been obtained by the use of crystal violet. This compound, a triphenylmethane dye has been described as effective against *T. cruzi* trypomastigotes in blood. Therefore, it has been used in some endemic areas in attempts to eliminate blood transmission of Chagas disease. Although it has been postulated to act through several mechanisms of action, crystal violet disrupts the Ca^2+^ homeostasis in *T. cruzi* epimastigotes and trypomastigotes, first by acting directly on the PMCA activity and its related Ca^2+^ transport, secondly dissipating the mitochondrial membrane potential with the concomitant release of Ca^2+^, and finally releasing Ca^2+^ from the endoplasmic reticulum (Docampo et al., 1993).

In addition, different treatments with distinct drugs are potentiated in the presence of Ca^2+^. For example, the effect of melarsoprol and also the combination of salicylhidroxamic acid with glycerol on African trypanosomiasis is significantly augmented when Ca^2+^ is present (Clarkson and Amole, 1982). Similarly, Ergosterone-coupled Triazol molecules trigger mitochondrial dysfunction, oxidative stress, and acidocalcisomal Ca^2+^ release in *Leishmania mexicana* (Figarella et al., 2015).

The anti-tuberculosis drug SQ109 (Figure 3), recently postulated as a promising candidate against resistant tuberculosis, and which is already in phase IIb-III clinical trials, acts on the lipid transporter MmpLs (Mycobacterial membrane proteins Large), which play crucial roles in transporting lipids, polymers and immunomodulators and which also extrude therapeutic drugs from the bacteria (Zhang et al., 2019). SQ109 has been recently investigated for activity against *T. cruzi*, where it was found to inhibit squalene synthase, responsible for a crucial step in ergosterol synthesis, and also to have a major effect causing parasite death through Ca^2+^ homeostasis, causing the collapse of the mitochondrial electrochemical potential, and impairing the function of acidocalcisomes (Veiga-Santos et al., 2015). Similar results were obtained in *L. mexicana* (García-García et al., 2016) and *L. donovani* (Gil et al., 2020), but interestingly demonstrating a more profound effect on amastigote-infected macrophages, the clinically relevant phase of the parasite life cycle, with IC_50_ found to be at the nanomolar range (IC_50_ ~7 nM).

An exception to the rule is Amphotericin B (AmB) a systemic antifungal once thought to exert its anti-parasitic effect through disruption of Ca^2+^ homeostasis. However, experimental evidence has demonstrated that although AmB is an efficient Ca^2+^ ionophore, the rapid permeabilization effect induced by AmB in Leishmania parasites is not dependent on an increase in [Ca^2+^]_i_, and is, at the same time, paradoxically enhanced by absence of external calcium (Cohen et al., 1990). Further, it has been demonstrated that, at low concentrations, AmB was able to form cation channels that collapsed the parasite membrane potential with no lytic effects; while, at high concentrations it provoked a salt influx via aqueous pores formation leading to osmotic changes inciting cell lysis (Ramos et al., 1996) and death of the parasite. In *T. cruzi*, AmB has shown to have a direct effect against all three developmental stages of the parasite, exhibiting a higher efficacy against the amastigote stage followed by the trypomastigote and epimastigote forms (De-Castro et al., 1993). This is important since it highlights the distinct susceptibility of vertebrate bound stages to this drug. To date, there is scarce information about the use of this polyene antifungal against *T. cruzi* in humans. However, its use in refractory cutaneous leishmania infections has been successfully documented (Morrison et al., 2010). There is insufficient evidence with which to make confident recommendations on the superiority in performance of conventional AmB deoxycholate over lipid-associated AmB compounds for treatment of trypanosomatid infections. However, liposomal AmB therapeutic failure has been reported for *Leishmania (L.) amazonensis* in humans (Morrison et al., 2010), and lack of efficacy has been demonstrated in murine models of acute and chronic *T. cruzi* infection (Clemons et al., 2017).

Another relevant drug against Chagas disease is the alkyl-lysophospholipid miltefosine (Figure 3), the only approved oral formulation against visceral leishmaniasis, the lethal form of the leishmania disease spectrum (Croft and Coombs, 2003), which has also shown promising anti-Trypanosomal activity (Luna et al., 2009; Saraiva et al., 2009; Rodriguez-Duran et al., 2019). Known mechanisms of action of miltefosine include inhibition of the synthesis of phosphatidylcholine, mitochondrial injury, and inhibition of the parasite cytochrome c oxidase (Pinto-Martinez et al., 2018b). In *T. cruzi*, this drug inhibits the biosynthesis of phosphatidylcholine 10–20 times more potently when compared to mammalian cells (Urbina, 2017). More recently, the spectrum of its mechanism of action has broadened, reporting a direct action on the disruption of the parasites Ca^2+^ homeostasis (Pinto-Martinez et al., 2018b; Rodriguez-Duran et al., 2019).

In *T. cruzi*, miltefosine is capable of opening a recently described sphingosine-activated plasma membrane Ca^2+^ channel (Rodriguez-Duran et al., 2019) which allows the opening of Ca^2+^ currents in a similar fashion to the physiological activator of the channel sphingosine. Concomitantly, miltefosine has also been demonstrated to collapse the mitochondrial electrochemical membrane potential (Δϕ), and to induce a rapid alkalinization of the parasites acidocalcisomes through direct action (Pinto-Martinez et al., 2018b). The synchronous action of miltefosine on Ca^2+^ permeability in the plasma membrane and membranes of intracellular organelles without affecting the human counterpart is to the best of our knowledge, a unique feature to this drug with vast potential beneficial effects for its use in humans.

Calcium channel blockers (CCB) have also shown promising therapeutic applications in trypanosomatid infections (Reimão et al., 2016). Many reports have demonstrated the action of Ca^2+^ channel antagonists from the L-type voltage-gated calcium channels VGCC as inhibitors of growth in different trypanosomatids (Tempone et al., 2009; De Rycker et al., 2016; Reimão et al., 2016; Kashif et al., 2017). Even though the substrate spectrum for action of calcium channel blockers in kinetoplastid parasites is predictably broad, it is very likely that these drugs directly act by activation of the aforementioned sphingosine-activated plasma membrane Ca^2+^ channel. If this were the case, aiming this channel as a potential anti-parasitic target would be an approach holding strong therapeutic implications. Further biophysical and biochemical characterization of this unique Ca^2+^-transporting system is needed for deciphering yet unresolved mechanisms within the parasites.

To date, several non-dihydropyridine calcium channel blockers (CCBs) have proved effective for inhibiting *in vitro* growth of *L. infantum* promastigotes and *T. cruzi* epimastigotes (Reimão et al., 2016), as well as by acting indirectly on reversing resistance against stibogluconate by mechanisms that remain yet to be elucidated (Neal et al., 1989). Despite some concerns regarding the use of non-dihydropyridines CCBs in heart failure due to their effect on reduction of cardiac contractility and reduction of heart rate and cardiac conduction (Abernethy and Schwartz, 1999), its longstanding use in humans for the treatment of hypertension and the ever-increasing evidence of these compounds on affecting and reducing parasitemia *in vitro* make them a promising group for drug repurposing against kinetoplastid parasites.

An abbreviate list of the most important functional targets of the main drugs affecting intracellular Ca^2+^ homeostasis of these parasites is depicted in Table 2.

**Table 2:** Targets of different drugs acting through disruption of Calcium homeostasis in different trypanosomatids.

**Drugs**	**Targets**	**References**
Amiodarone	Mitochondria, Acidocalcisomes, Ergosterol synthesis	Benaim et al., 2006; Serrano-Martín et al., 2009a,b; Benaim and Paniz-Mondolfi, 2012
Dronedarone	Mitochondria, Acidocalcisomes, Ergosterol synthesis	Benaim and Paniz-Mondolfi, 2012; Benaim et al., 2012, 2014
SQ109	Mitochondria, Acidocalcisomes, Ergosterol synthesis	Veiga-Santos et al., 2015; García-García et al., 2016; Gil et al., 2020
Amioder (Benzofuran derivative)	Mitochondria, Acidocalcisomes,	Pinto-Martinez et al., 2018a; Martinez-Sotillo et al., 2019
Miltefosine	Sph-activated Plasma membrane Ca^2+^-Channel, Mitochondria, Acidocalcisomes	Pinto-Martinez et al., 2018b; Rodriguez-Duran et al., 2019
Posaconazole	Elevation of intracellular Ca^2+^	Benaim et al., 2006

## Concluding Remarks

The identification of new intracellular calcium-linked anti-parasitic targets is rapidly expanding the set of potential therapeutic options against trypanosmatid infections. Because target-based toxicity and side effects may arise due to cross reactivity with human homologues caution is advised when looking at structural differences between species while preferably choosing exquisitely selective anti-parasitic inhibitors. Many of these compounds are already a relevant part of the current clinical arsenal to treat Chagas disease and are likely to remain so for the foreseeable future; examples include the antiarrhythmic agent amiodarone and emerging benzofuran derivatives, as well as the calcium channel blockers. Others, such as the azole derivatives (e.g., posaconazole) and the new antituberculosis drug SQ109, are gaining relevance as they are repurposed as anti-parasitic drugs. An increasing body of experimental evidence supports the disruption of parasite Ca^2+^ homeostasis and intracellular Ca^2+^ storage compartments as strategic targets for treatment of trypanosomatids infections in humans.

## Author Contributions

GB conceive and wrote the review. AP-M, ES, and NM-S participate directly in the writing of the review.

### Conflict of Interest

The authors declare that the research was conducted in the absence of any commercial or financial relationships that could be construed as a potential conflict of interest.
